# Dendritic Cell Activating Receptor 1 (DCAR1) Associates With FcεRIγ and Is Expressed by Myeloid Cell Subsets in the Rat

**DOI:** 10.3389/fimmu.2019.01060

**Published:** 2019-05-10

**Authors:** Michael R. Daws, Britt Nakken, Ana Lobato-Pascual, Régis Josien, Erik Dissen, Sigbjørn Fossum

**Affiliations:** ^1^Department of Molecular Medicine, Institute of Basic Medical Sciences, University of Oslo, Oslo, Norway; ^2^Centre de Recherche en Transplantation et Immunologie UMR 1064, INSERM, Université de Nantes, Nantes, France; ^3^Laboratoire d'Immunologie, CHU Nantes, Nantes, France

**Keywords:** macrophage, granulocyte, dendritic cell, C-type lectin, receptor

## Abstract

Dendritic cell activating receptor-1 (DCAR1) is a cell-surface receptor encoded by the Antigen Presenting Lectin-like gene Complex (APLEC). We generated a mouse monoclonal antibody against rat DCAR1, and used this to characterize receptor expression and function. Rat DCAR1 was expressed on minor subsets of myeloid cells in lymphoid tissue, but was uniformly expressed at a high level by eosinophils, and at a low level by neutrophils. It was expressed by eosinophils in the peritoneal cavity and the lamina propria of the gut, and by subsets of macrophages or dendritic cells at these sites. Polarization of peritoneal macrophages showed that DCAR1 expression was absent on M1 macrophages, and increased on M2 macrophages. DCAR1 could be expressed as a homodimer and its associated with the activating adaptor protein FcεRIγ. This association allowed efficient phagocytosis of antibody-coated beads. Additionally, cross-linking of DCAR1 on the surface of rat eosinophils lead to production of reactive oxygen species. These data show that DCAR1 is an activating receptor. Its expression on M2 macrophages and eosinophils suggests that it may play a role in the immune response to parasites.

## Introduction

We have previously identified a phylogenetically conserved cluster of structurally related receptor genes expressed primarily by myeloid cells (1). The complex, which we named the antigen-presenting cell lectin-like receptor gene complex (APLEC), encodes receptors belonging to group II C-type-lectins, i.e., type II surface receptors containing a single C-type lectin domain (CTLD) that retains the calcium-coordinating residues necessary for saccharide-binding (2). Whereas, the human APLEC contains five functional genes (DLEC, DCIR, Dectin 2, MCL, and Mincle), the mouse encodes nine and the rat seven genes presumed to be functional (DCAR1, MCL, Mincle and DCIR1,−2,−3, and−4). In contrast to the mouse APLEC, which encodes two DCAR family members (DCAR1 and−2, the latter also called DCAR), the rat DCAR family consists of only one intact gene, *Dcar1*, with *Dcar2* reduced to incomplete gene fragments. There is no direct human ortholog of DCAR1, although we have previously suggested that DLEC may fill this role (1).

The APLEC region is associated with rheumatoid arthritis in man and with susceptibility to experimentally induced autoimmunity in rodents (3–5). Prime examples are oil-induced arthritis (OIA) (3) and experimental allergic encephalomyelitis (EAE) (5), which represent experimental models for rheumatoid arthritis and multiple sclerosis, respectively. In the rat, the inbred DA strain is OIA- and EAE-sensitive, while the PVG strain is resistant. For both traits association of disease susceptibility to the APLEC has been shown by the transfer of the APLEC region from the PVG strain through back-crossing into the genetic background of the DA strain. DA.APLEC^PVG^ congenic rats are resistant to OIA and EAE (3, 5). The DA.APLEC^PVG^ congenic rats also differ from DA rats with respect to reactivity to infectious diseases (6). On this background the *Dcar1* gene is of particular interest, because the DA allele carries a nonsense mutation in the second exon (encoding the transmembrane domain) that prevents productive expression of DCAR1 protein at the cell surface (1).

In the mouse, DCAR1 has been shown to be expressed on subsets of myeloid cells, including CD8^+^ dendritic cells (7). Antibody to mouse DCAR1 could deliver antigen to CD8^+^ DCs *in vivo* and stimulate proliferation of T cells; T cell production of IL-12 increased while production of IL-10 decreased, suggesting Th1-polarization of the immune response (7). Although the signaling properties of mouse DCAR1 were not studied, close sequence similarity to the DCAR2 paralogue, shown to mediate activating signals through its association with the FcεRIγ signaling adaptor (8), suggests that DCAR1 is also an activating receptor.

Here we have developed a monoclonal antibody to rat DCAR1, and used this to characterize the biochemistry and expression of the receptor in the rat. We show that rat DCAR1 is expressed on subsets of myeloid cells in several tissues, being particularly prominent in the peritoneal cavity and the lamina propria of the gut. We further show that rat DCAR1 associates with the FcεRIγ signaling adaptor and that this complex mediates phagocytosis of antibody-coated beads. Additionally, cross-linking of DCAR1 on the surface of freshly isolated eosinophils leads to production of reactive-oxygen species (ROS). Our findings for rat DCAR1 confirm previous observations in the mouse, but suggest fundamental differences in expression and function between the two species.

## Materials and Methods

### Animals

Animals were reared under conventional conditions in individually ventilated cages at the Institute of Basic Medical Sciences (Domus Medica), University of Oslo. DA.APLEC^PVG^ (9) and DA.NKC^PVG^ (10) congenic rats were generated and maintained in the same animal facility; the two strains were derived from a larger chromosome-4 congenic rat strain by back-crossing to DA rats, and they retain the PVG APLEC or NKC gene complexes, respectively (DA.APLEC^PVG^ express DCAR at the cell surface while DA.NKC^PVG^ do not due to a mutation in the DA DCAR allele). BALB/c mice were purchased from Harlan. Animals were terminated by CO_2_ narcosis. This study was carried out in accordance with the recommendations of The European Union's Directive 2010/63/EU on the protection of animals used for scientific purposes. The protocol was approved by the Norwegian Food Safety Authority.

### Cell Lines and Primary Cells

Cell lines were obtained from ATCC (Manassas,VA) and were grown in RPMI 1640 supplemented with 10% heat-inactivated FCS and 1% antibiotic-antimycotic (Invitrogen), hereafter termed complete RPMI.

Blood was collected from the right atrium in heparinized syringes (heparin from LEO Pharma). Erythrocytes were removed by hypotonic lysis (ACK buffer: 0.15 M NH_4_Cl, 10 mM KHCO_3_, 0.1 mM Na_2_EDTA). Single-cell suspensions were prepared from spleen and lymph nodes by first collecting the organs on ice-cold PBS, cutting them into small pieces and digesting with 2 μg/ml collagenase D (Roche) in RPMI 1640 with 1% FCS for 30 min at 37°C. The digestion was stopped with 10 mM EDTA and tissue was filtered through a 70 μm cell strainer (BD Biosciences). Peritoneal cavity cells were collected by peritoneal lavage using ice-cold PBS. Lamina propria cells were isolated from the small intestine. Briefly, the whole small intestine was taken out and flushed with ice cold PBS. Sections containing Peyer's patches were removed, and the remaining parts were cut into 2 cm pieces and inverted. The pieces were then incubated in PBS/1% BSA/2 mM EDTA/0.3 mg/ml DTT for 15 min at 37°C with vigorous shaking and supernatant containing shed epithelial cells was removed. This step was repeated three times before the remaining tissue was rinsed with PBS and digested in RPMI containing 2 μg/ml collagenase D and 40 μg/ml DNAse I for 2x 20 min at 37°C with shaking. Cells recovered from the digest were washed in PBS and filtered through a 70 μm cell strainer.

### Polarization of Peritoneal Macrophages

Peritoneal cavity cells were collected by peritoneal lavage using ice-cold PBS. Cells were counted and resuspended in phenol red-free DME/F12 medium supplemented with 10% FCS, and 1% antibiotic-antimycotic at 1 × 10^6^ cells/ml. 5 × 10^6^ cells were plated per well in non-tissue culture-treated 6-well plates, and left to adhere for 2 h at 37°C, before washing 5 times with PBS to remove non-adherent cells. Cells were then incubated overnight in medium alone (M0), or supplemented with 100 ng/ml IFN-γ and 10 ng/ml LPS (M1), or with 50 ng/ml IL-13 and 20 ng/ml IL-4 (M2). Cells were removed from the wells using PBS with 10 mM EDTA and 0.5% FCS. For sorting experiments, cells were labeled with OX42 and OX41 antibodies, and OX42 hi and lo populations sorted on a FACS Aria cell sorter. Sorted cells were lysed and total RNA prepared using NucleoSpin RNA kit (Machery-Nagel).

### Real-Time PCR

First strand cDNA synthesis was performed using Superscript III system (Thermo Fisher) and oligo dT primer, according to the manufacturer's instructions. Realtime PCR reactions were carried out in 384 well plates using SSoAdvanced SYBR Green supermix (BioRad) and PCR performed in an ABI 7900 HT machine. The following rat-specific primer sets were used: ACTB-F: AAGTCCCTCACCCTCCCAAAAG, ACTB-R: AAGCAATGCTGTCACCTTCCC; CCR7-F: CGAGCCTTCCTGTGTGACTT, CCR7-R: CCACCACCAGCACGTTTTTC; CLEC10A-F: AGGGAAGCCACGATTTCACA, CLEC10A-R: TACTGAGCTGGGACCAAGGA; MRC1-F: AACTGCGTGGATCCCTTTCC, MRC1-R: CTCGATGGAAACCAGGGAGG; IL1β-F: GCAACTGTCCCTGAACTCAA, IL1β-R: TGTCAGCCTCAAAGAACAGG; TNFα-F: CAGACCCTCACACTCAGATCA, TNFα-R: TTGTCTTTGAGATCCATGCC; NOS2-F: GAGTTCACCCAGTTGTGCAT, NOS2-R: GCACAAGATCAGGAGGGATT.

### Expression Constructs

Full-length open reading frames of rat DCAR1 or DCIR1 were cloned into a pEMCV-SRα expression vector containing a C-terminal FLAG tag. Full length open reading frames of DAP10, DAP12, FcεRIγ, and CD3ζ signaling adaptor proteins were cloned into pDISPLAY expression vectors (Life Technologies) containing an N-terminal HA-tag. For testing the antibody, chimeric cDNAs were constructed by cloning the extracellular domains of the APLEC receptors into a pMXpuro vector containing the CD3ζ cytoplasmic domain, the transmembrane domain of KLRH1 and a C-terminal FLAG-tag—these chimeric constructs allow high level expression in the absence of signaling adaptors. Expression constructs were used for the transient transfection of 293T cells using polyethyleneimine “Max” MW 25,000 (Polysciences) (11).

### Production of a Monoclonal Antibody Toward Rat DCAR1

A soluble fusion protein consisting of the extracellular domain of rat DCAR1 and the Fc region of mouse IgG2b was used for the intraperitoneal immunization of mice. Splenocytes were fused with NS0 myeloma cells using standard techniques, and supernatants were screened for binding to DCAR1-transfected 293T cells. Five positive hybridomas (WEN37-41) were subcloned and expanded. Of these, mAb WEN41 (isotype IgG1) proved most versatile as it not only showed specific and high-affinity binding to DCAR1 on rat primary cells, but also worked in western blotting and immunohistochemistry.

### Flow Cytometry

The following commercial antibodies were used: anti-CD3 (G4.18), anti-CD161 (10/78), anti-CD45R (OX33), and anti-CD103 (OX62) (all BD Biosciences); anti-TCRγδ (V65) and anti-CD4 (W3/25) (Biolegend); anti-MHC class II (OX17, eBioscience); anti-FLAG (M2, Sigma); and anti-HA (HA11, Covance). Anti-TCRαβ (R73) and anti-CD11b/c (OX42) were produced in-house. The 85C7 antibody has previously been described (12). Single-cell suspensions were prepared and 1 × 10^6^ cells were incubated for 15 min in 50 μl PBS/1% FCS/10% rat serum/10 mM NaN_3_. The appropriate mAbs were added in 50 μl PBS/1% FCS/NaN_3_ and incubated for 20 min at 4°C. Data were acquired using a FACSCalibur or FACSCanto II flow cytometer (BD Biosciences) and analyzed using FlowJo software (Tree Star). Lamina propria and peritoneal cavity cell suspensions were filtered and sorted on a FACSAria II (BD Biosciences) at 20 psi using a 100 μm nozzle. Cell aggregates and dead cells were excluded from analysis prior to sorting. Cells were sorted at 4°C and collected into low-binding falcon tubes (BD Biosciences) containing RPMI 1640/10% FCS. Sorted cells were immediately processed for cytospin preparations.

### Cytospin and Immunohistochemistry

Lamina propria and peritoneal cavity cell suspensions were spun onto poly-L-lysine coated slides for 8 min at 800 rpm in a Cytospin 2 centrifuge (Shandon). Slides were fixed in methanol and dried before staining with May-Grünwald-Giemsa. Tissue samples were snap frozen in isopentane cooled in liquid nitrogen and mounted on chucks in embedding media (NEG-50, Thermo Scientific). Frozen small intestine sections (5–10 μm) were fixed in acetone, washed in PBS, incubated with hydrogen peroxidase (Hydrogen peroxidase block, Thermo Scientific) to block endogenous peroxidase activity, washed 3 x in PBS and then in PBS containing 10% non-immune rabbits serum, and then incubated with primary antibodies. Peroxidase-conjugated rabbit anti-mouse IgG (Sigma-Aldrich) was pre-absorbed against tissue homogenates from rat lymph nodes and used as secondary antibody diluted 1:1000 in PBS with 2% rat serum. Peroxidase activity was demonstrated with 3-3-diaminobenzidine (UltraVision detection system, Thermo Scientific).

### Western Blot Analysis

Transfected 293T cells were lysed in cold lysis buffer containing 50 mM n-octyl β-D-glucopyranoside and 0.1% Igepal CA-630 (Sigma-Aldrich), 25 mM Tris-HCl, pH 7.5, 150 mM NaCl, 1X Halt Protease and Phosphatase Inhibitor Cocktail, EDTA-free (Thermo Fisher Scientific) in the presence of 0.1 mM CaCl_2_ and 1 mM MgCl_2_. Lysates were immunoprecipitated with anti-DCAR1 mAb WEN41, or with a mouse IgG1 isotype control antibody, before separation by SDS-PAGE under non-reducing and reducing (+50 mM DTT) conditions, respectively. Following transfer to polyvinylidene difluoride membranes (Immobilon P, Millipore), bands were detected with anti-HA or anti-FLAG antibodies followed by goat anti-mouse IgG-HRP (Jackson ImmunoReseach Laboratories), and blots developed using Supersignal West Pico Chemiluminescent Substrate (Thermo Fisher Scientific) and Hyperfilm-ECL (GE Healthcare Life Sciences).

### Phagocytosis Assay

293T cells were transiently transfected with constructs encoding DCAR1-FLAG alone or together with constructs encoding each of the adaptor protein constructs DAP10, DAP12, FcεRIγ, and CD3ζ. Transfection efficiency was tested by flow cytometry. 293T cells were incubated with Neutravidin-coupled yellow-green fluorescent 1 μm microspheres (Thermo Fisher) coated with DCAR1 antibody, counterstained with streptavidin-DyLight594 and analyzed by imaging flow cytometry on an ImageStream X (Amnis) as described previously (13). To compare efficiency of phagocytosis, data are expressed as phagocytic ratio: (fraction of bead-binding cells with internalized beads)/(fraction of bead-binding cells with no internalized beads). Data were analyzed with Graphpad Prism software using one-way ANOVA, followed by Dunnet's multiple comparison test.

### ROS Assay

Ninety-six-well plates were coated with anti-mouse IgG at 10 μg/ml overnight at 4°C, washed 3 times in PBS, blocked in PBS/1% BSA for 1 h at 37°C and then incubated with F(ab')_2_ anti-DCAR1 antibody or isotype control for 1 h. Control wells were not coated. Plates were washed 3 times with PBS before use. Peritoneal cells were isolated by rinsing the peritoneal cavity with PBS. Recovered cells were resuspended in complete RPMI at 2 × 10^6^ cells/ml, and then incubated in tissue culture flasks for 90 min at 37°C to remove adherent cells. 1 × 10^5^ enriched cells were transferred to each well and incubated for 15 min at 37°C, before dihydrorhodamine 123 was added to a final concentration of 5 μg/ml, and incubated for a further 5 min before cells were fixed by adding an equal volume of PBS/4% paraformaldehyde. Production of ROS by eosinophils was evaluated by flow cytometry, gating eosinophils by forward, and side scatter. Data were analyzed with Graphpad Prism software using the unpaired *t*-test, and statistical significance determined using the Holm-Sidak method, with alpha = 0.05.

## Results

### Generation of Anti-rat DCAR1 Antibody

**Figure 1:**
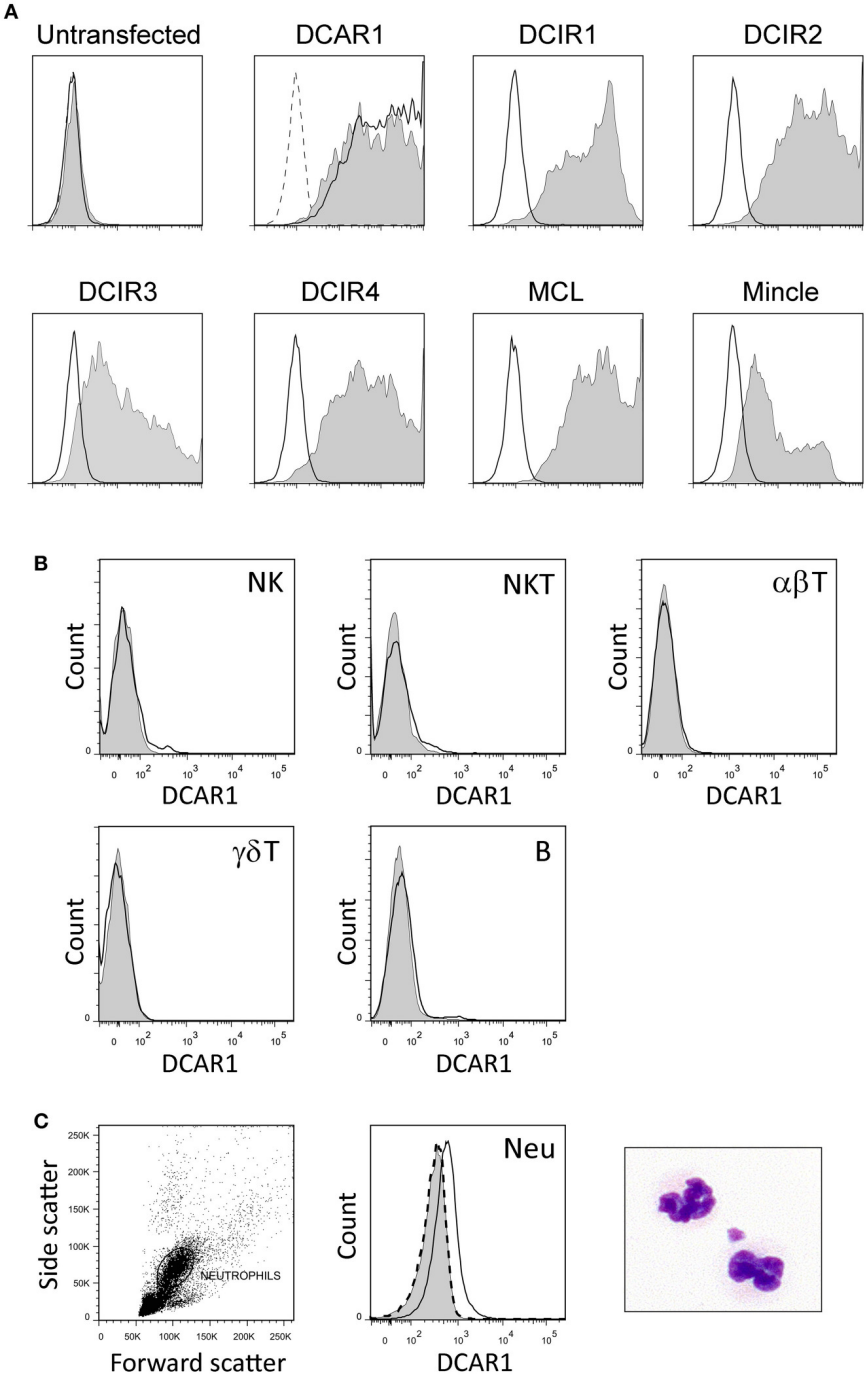
Characterization of a novel monoclonal antibody toward rat DCAR1, and flow cytometry analysis of blood and spleen leukocytes. **(A)** Untransfected 293T cells or 293T cells transfected with chimeric constructs containing the extracellular domains of the indicated APLEC receptors were analyzed by flow cytometry using mAb toward the FLAG epitope (filled curves) or to DCAR1 (WEN41, solid lines). Staining with isotype antibody (dashed lines) is shown on the first two plots, but omitted from the others for the sake of clarity. **(B,C)** Subsets of leukocytes from DA.APLEC^PVG^ congenic rats were identified using specific markers, and DCAR1 expression analyzed using mAb WEN41 (solid lines) or isotype control (filled curves). WEN41 staining of DA.NKC^PVG^ congenic rats (DCAR1-deficient) is also shown as a control (dashed line). **(B)** Splenic lymphoid populations. **(C)** Neutrophils (Neu).

Mice were immunized with rat DCAR1-Fc fusion protein, and hybridomas screened for reactivity against transfected cells. A specific monoclonal antibody, WEN41, was generated and used for the studies described here. This mAb bound specifically to DCAR1-transfected 293T cells, but not to untransfected cells, or to cells transfected with other APLEC receptors (Figure 1A).

### DCAR1 Is Expressed on Subsets of Myeloid Cells in Blood and Spleen

Immunohistochemistry of spleen cryosections showed only weak staining with WEN41. Correspondingly, flow cytometry analysis showed no DCAR1 expression on lymphocytes in the blood or spleen (Figure 1B and data not shown). Uniform and reproducible, but relatively low expression of DCAR1 was also detected on blood neutrophils (Figure 1C).

**Figure 2:**
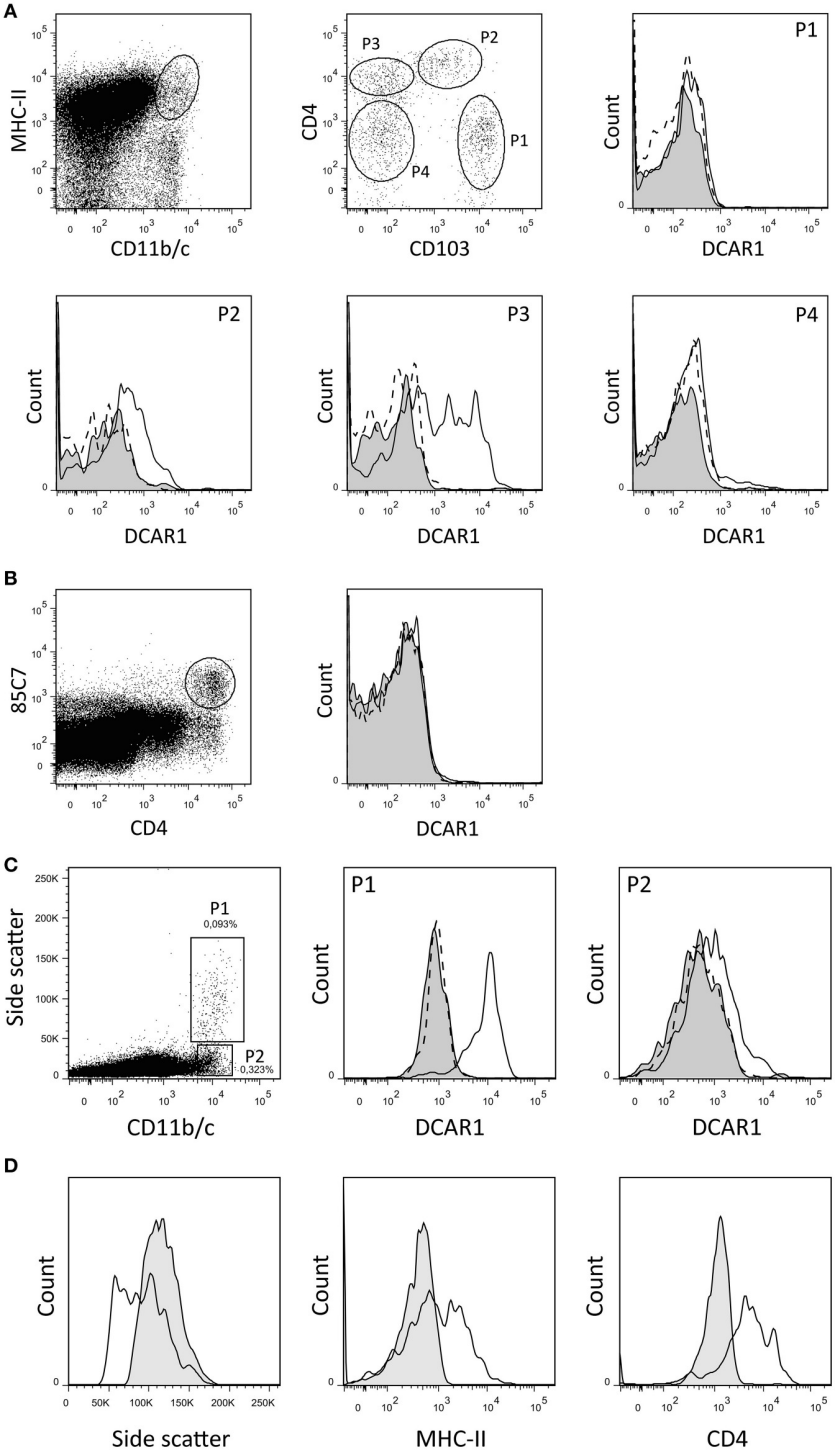
Flow cytometry analysis of splenic dendritic cells and mesenteric lymph node leukocytes. **(A,B)** DC subsets from DA.APLEC^PVG^ congenic rats were identified using the indicated gating strategy, and DCAR1 expression analyzed using WEN41 (solid lines) or isotype control (filled curves). WEN41 staining of DA.NKC^PVG^ congenic rats (DCAR1-deficient) is also shown as a control (dashed line). **(A)** Conventional DC. **(B)** pDC. **(C)** CD11b/c^hi^ cells from mesenteric lymph nodes from DA.APLEC^PVG^ congenic rats could be split into two subsets on the basis of side-scatter. DCAR1 expression was analyzed on these subsets using mAb WEN41 (solid lines) or isotype control (filled curves). WEN41 staining of DA.NKC^PVG^ congenic rats (DCAR1-deficient) is also shown as a control (dashed line). **(D)** Comparison of the P1 subset (solid line) to blood eosinophils (filled curves).

By gating splenocytes on CD11b/c^hi^ MHC II^hi^ cells, four minor populations could be distinguished on the basis of CD103 and CD4 expression (Figure 2A). P1 and P2 correspond to two populations of conventional DC that were previously identified, and that probably represent cDC1 and cDC2 populations, respectively (14). The P1 (cDC1) subset did not express DCAR1. In contrast, the P2 (cDC2) subset showed robust expression of DCAR1. Additionally, a CD103^−^CD4^+^ subset of cells (P3) showed heterogeneous expression of DCAR1, with some cells having very high DCAR1 expression (Figure 2A). In the rat, plasmacytoid dendritic cells are defined by high CD4 expression and reactivity with the 85C7 mAb (12). No expression of DCAR1 could be detected on CD4^hi^85C7^+^ plasmacytoid DC (Figure 2B).

### High DCAR1 Expression on Subsets of Myeloid Cells in Mesenteric Lymph Nodes

In the mesenteric lymph nodes, DCAR1 expression could be detected on a population of CD11b/c^hi^ cells. These cells could be separated into two subpopulations on the basis of side scatter (Figure 2C). The P2 population most likely represents a subset of DCs, and these showed low DCAR1 expression. The P1 population expressed high levels of DCAR1 on the cell surface. These may represent eosinophils that have traveled to the lymph nodes from the gut, but they differed from blood eosinophils in regards to side scatter as well as MHC-II and CD4 expression (Figure 2D).

### DCAR1 Is Highly Expressed on Eosinophils in the Peritoneal Cavity

**Figure 3:**
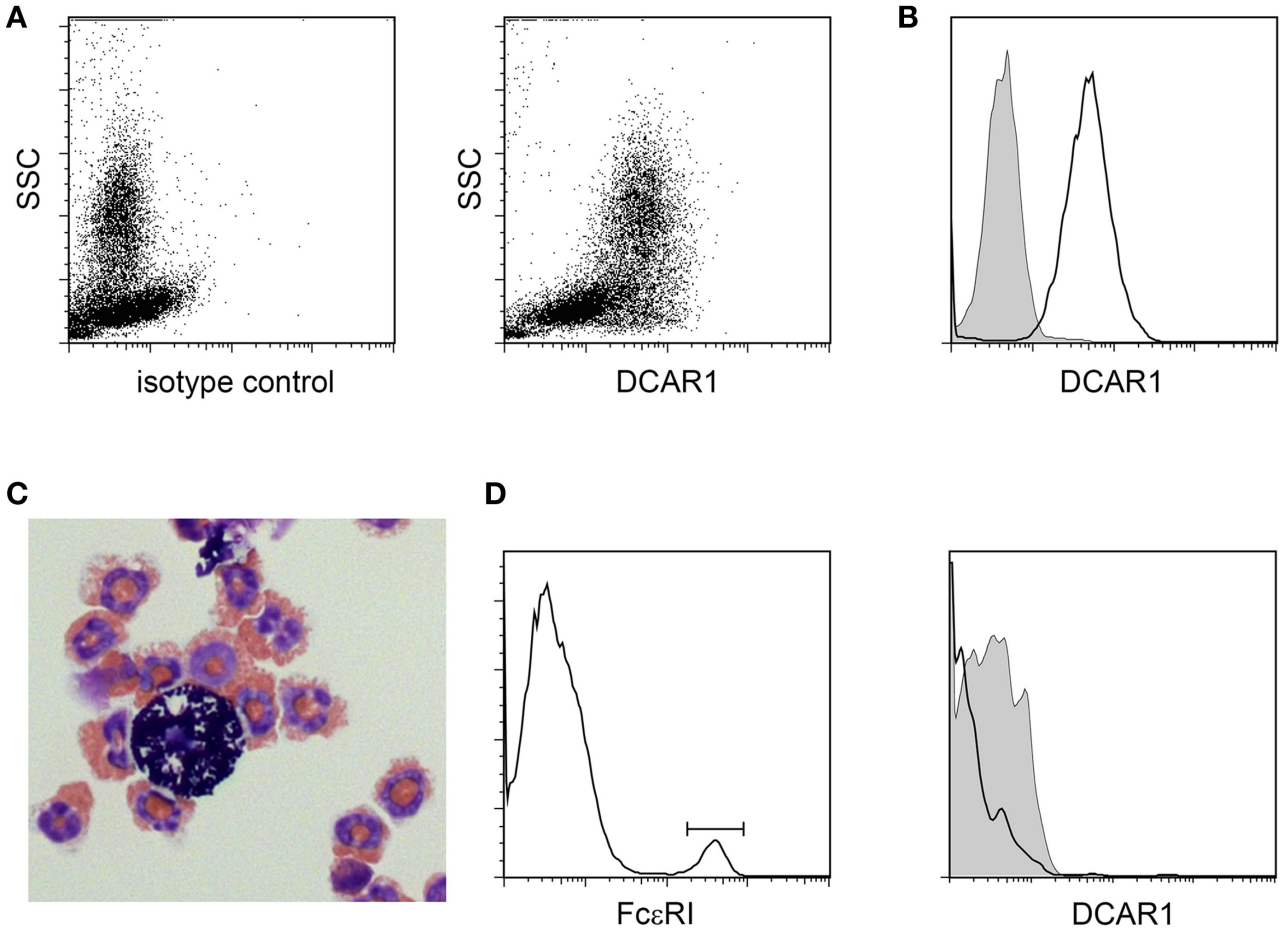
Analysis of peritoneal leukocytes. **(A)** Identification of a high side-scatter population with high DCAR1 expression. **(B)** DCAR1 expression of cells gated for high side-scatter. **(C)** Cytospin analysis of sorted high side-scatter cells. **(D)** Analysis of DCAR1 expression on mast cells.

In the peritoneal cavity a clear population with high side scatter could be discerned that expressed high levels of DCAR1 (Figures 3A,B). To investigate the morphology of these cells, SSC^hi^DCAR1^+^ cells were sorted by FACS and prepared as cytospins. May-Grunwald-Giemsa staining revealed that the sorted cells consisted mainly of eosinophils, together with a smaller population of mast cells (Figure 3C). SSC high, FcεRI^+^ cells did not show expression of DCAR1 (Figure 3D), indicating that the DCAR1^+^ SSC^hi^ cells represented eosinophils and not mast cells.

### DCAR1 Is Preferentially Expressed on M2 Macrophages

**Figure 4:**
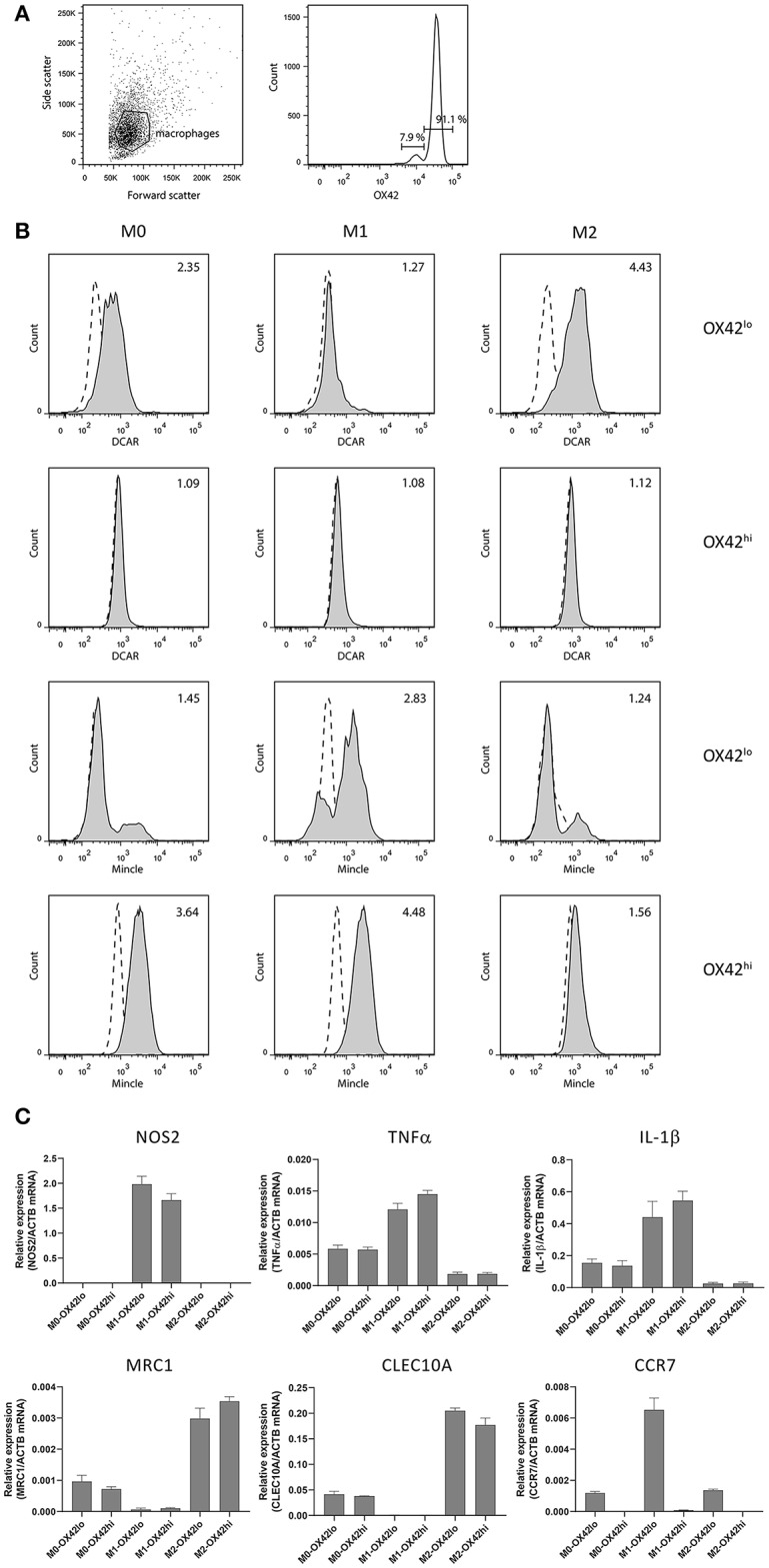
Analysis of polarized peritoneal macrophages. **(A)** OX42-staining of peritoneal macrophages purified by adhesion and cultured overnight. **(B)** Staining of OX42hi and OX42lo macrophages with antibodies against Mincle or DCAR1, following overnight culture under polarizing conditions. **(C)** Real-time PCR analysis of polarized and OX42-sorted populations assessing expression of typical M1 and M2 polarization markers.

In addition to the high SSC population in the peritoneal cavity, a lower SSC population could also be discerned corresponding to peritoneal macrophages. We isolated these cells by adhesion to tissue culture plates, and then maintained them overnight without stimulation (M0), or polarized to an M1 phenotype with IFNγ + LPS, or toward an M2 phenotype with IL-4 + IL-13. Two populations of macrophages could be distinguished based on their expression of CD11b/c (OX42) (Figure 4A). These populations most probably correspond to populations previously described in the mouse, where a major CD11b^hi^ population represents resident macrophages, while a minor CD11b^lo^ population represents migratory macrophages derived from blood monocytes (15). Real-time PCR on polarized populations sorted based on OX42 expression showed that both populations were appropriately polarized. Thus M1 polarized macrophages showed upregulation of NOS2, TNF-α and IL-1β, while M2 polarized macrophages upregulated mannose receptor (MRC1) and CLEC10A. Strikingly, CCR7, a typical M1 marker, was present only on OX42^lo^ macrophages, although upregulated on the M1-polarized OX42^lo^ population. This is consistent with the idea that these are migratory macrophages, as in the mouse (Figure 4C). Additionally, only this OX42^lo^ population regulated expression of DCAR1 on the cell surface upon M2 polarization. Both populations upregulated Mincle upon M1 polarization, a receptor known to be inducible by LPS and IFN-γ (16). Thus, M1-polarized macrophages had no expression of DCAR1 on the cell surface, but upregulated expression of Mincle. Conversely, M2-polarized macrophages did not upregulate Mincle, but increased expression of DCAR1 (Figure 4B).

### DCAR1 Expression in Diffuse Mucosa-Associated Lymphoid Tissues

**Figure 5:**
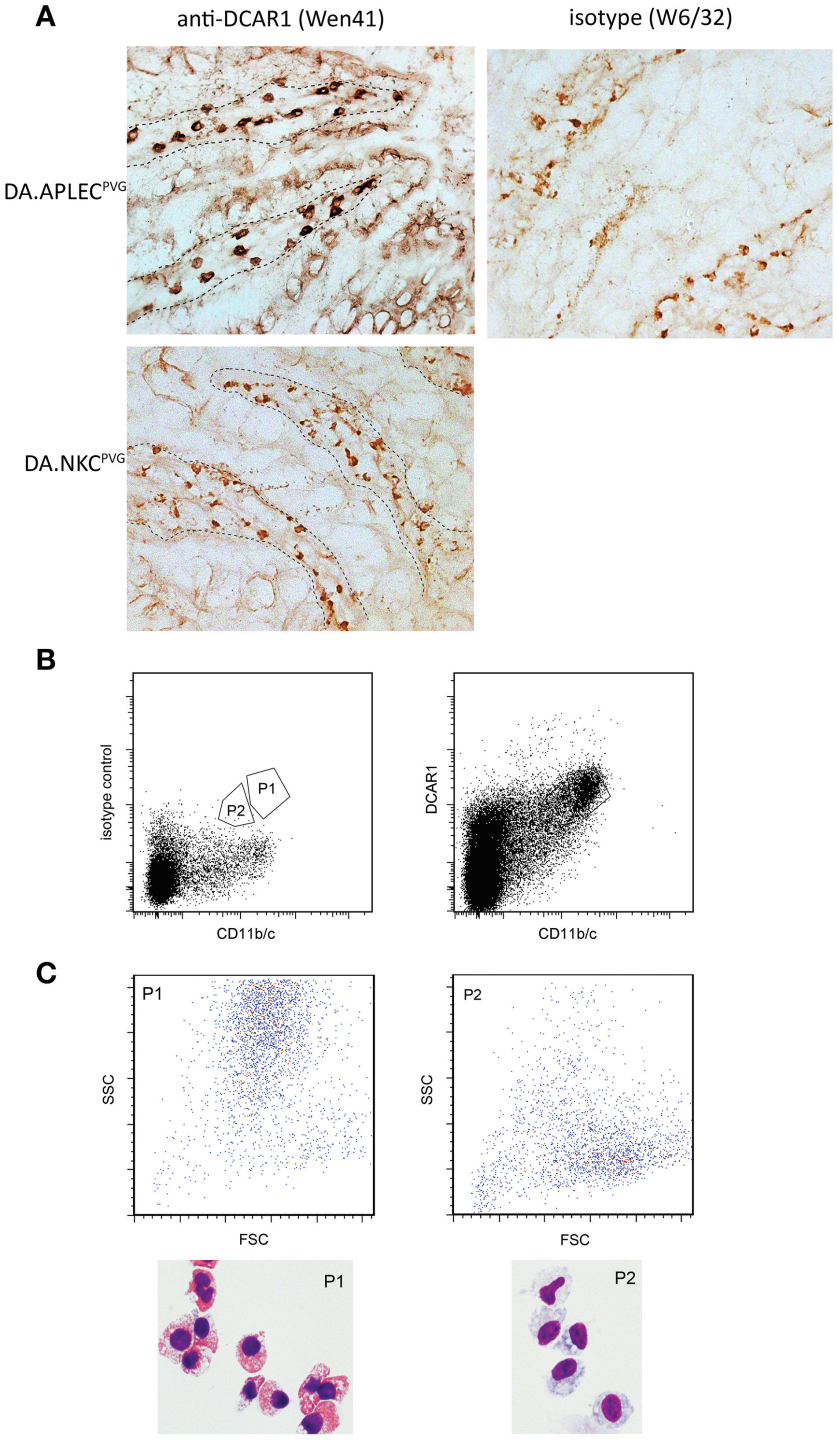
Analysis of gut leukocytes. **(A)** Immunohistochemical staining of cryosections from the small intestine. Staining with WEN41 on tissue from DA.APLEC^PVG^ rats and DA.NKC^PVG^ rats. Boundary between epithelium and the lamina propria is indicated with a dashed line. In addition to tissue from DA rats, negative controls included omission of primary antibody (not shown). Non-specific peroxidase staining, probably reflecting endogenous eosinophil peroxidase activity, is observed in the control sections, but is easily distinguishable from the specific staining be being weaker and displaying a granular cytoplasmic appearance. **(B)** Sorted populations of DCAR1+ cells from the lamina propria. **(C)** Analysis of sorted populations for forward and side scatter, and cytospin staining of isolated cells.

**Figure 6:**
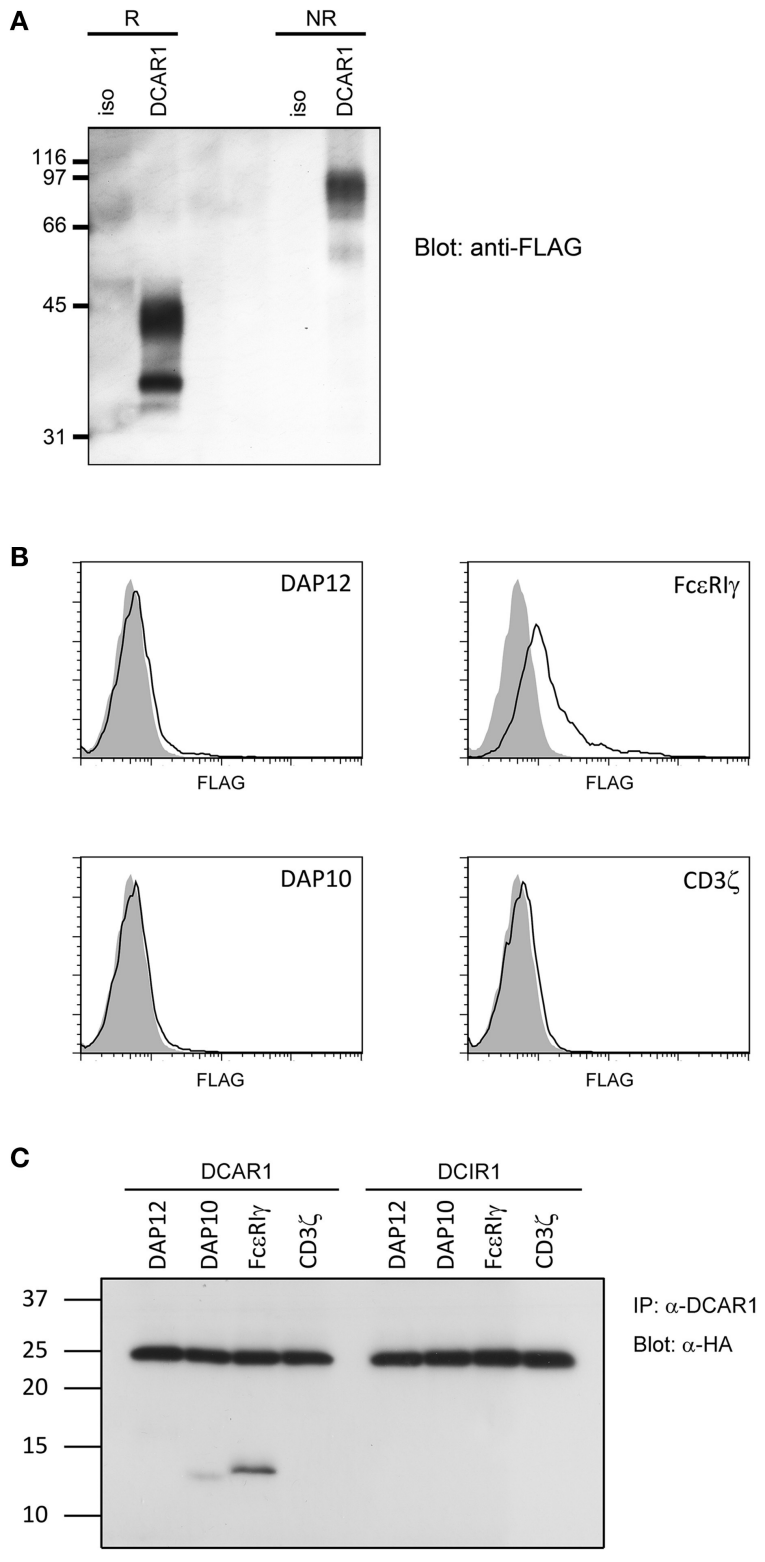
Biochemical analysis of rat DCAR1. **(A)** Lysates from 293T cells transfected with rat DCAR1-FLAG were immunoprecipitated with mAb WEN41 and subjected to western blot analysis with an anti-FLAG antibody under reducing (R) or non-reducing (NR) conditions. **(B)** Flow cytometric analysis of DCAR1 surface expression following transfection of 293T cells with DCAR1-FLAG alone (filled curves) or together with the indicated adaptor proteins (solid lines). **(C)** Lysates from 293T cells transfected with DCAR1 or DCIR1 together with the indicated HA-tagged adaptor proteins were immunoprecipitated with mAb and subjected to western blot analysis with an anti-HA antibody. Relative molecular mass in kDa is indicated **(A,C)**.

Immunohistochemical staining of sections from the small intestine revealed large numbers of cells in the lamina propria of the gut that showed strong WEN41 staining of the plasma membrane (**Figure 6A**, left panel). This was not seen in the gut of the DCAR1-deficient DA.NKC^PVG^ rat strain, nor on cells stained with isotype control antibody (Figure 5A, right panel and data not shown). A weaker cytoplasmic staining could be seen in some cells, which probably represents residual endogenous peroxidase activity present in eosinophil granules. In contrast, immunostaining of lungs showed extremely few/no DCAR1+ cells (Figure S1).

We isolated cells from the gut mucosa and used FACS sorting to separate out two populations that showed high expression of DCAR1 and CD11b/c (Figure 5B). Cytospins were made with the two sorted subsets. Morphologically, the CD11b/c^hi^DCAR1^+^ subset corresponded to eosinophils (Figure 6C, P1) whereas the subset of DCAR^+^ cells with lower expression of CD11b/c had a typical macrophage-like morphology (Figure 5C, P2).

### DCAR1 Is a Disulfide-Linked Dimer

Rat DCAR1 consists of 208 amino acid residues and has a predicted molecular weight of 24.3 kDa. The amino acid sequence contains three putative N-glycosylation sites (1). Transiently transfected 293T cells with DCAR1-FLAG expression constructs were lysed and lysates separated by SDS-PAGE under non-reducing and reducing conditions. Western blot analysis with an anti-FLAG antibody detected a major band of ~42 kDa under reducing conditions, and approximately 90 kDa under non-reducing conditions (Figure 6A), indicating that DCAR1 was expressed as a disulfide-linked homodimer.

### DCAR1 Associates With FcεRIγ

Rat DCAR1 contains a positively charged residue in its putative transmembrane domain, suggesting that it may associate with a signaling adaptor protein. To investigate this possibility, we transfected 293T cells with DCAR1 alone, or DCAR1 together with the adaptor proteins DAP10, DAP12, FcεRIγ, or CD3ζ. Only FcεRIγ could increase expression of DCAR1 on the cell surface, suggesting that this is the adaptor that forms a functional complex with DCAR1 (Figure 6B). To confirm this association, 293T cells were transfected with DCAR1 or DCIR1 together with each of the four adaptor proteins, and then immunoprecipitated with WEN41 mAb. FcεRIγ was readily precipitated when co-transfected with DCAR1, but not with DCIR1, suggesting a specific association between rat DCAR1 and FcεRIγ (Figure 6C). Despite its inability to induce DCAR1 expression on the cell surface, DAP10 could also be reproducibly co-precipitated with DCAR1 although always to a much lower degree than FcεRIγ.

### DCAR1/FcεRIγ Mediates Phagocytosis

**Figure 7:**
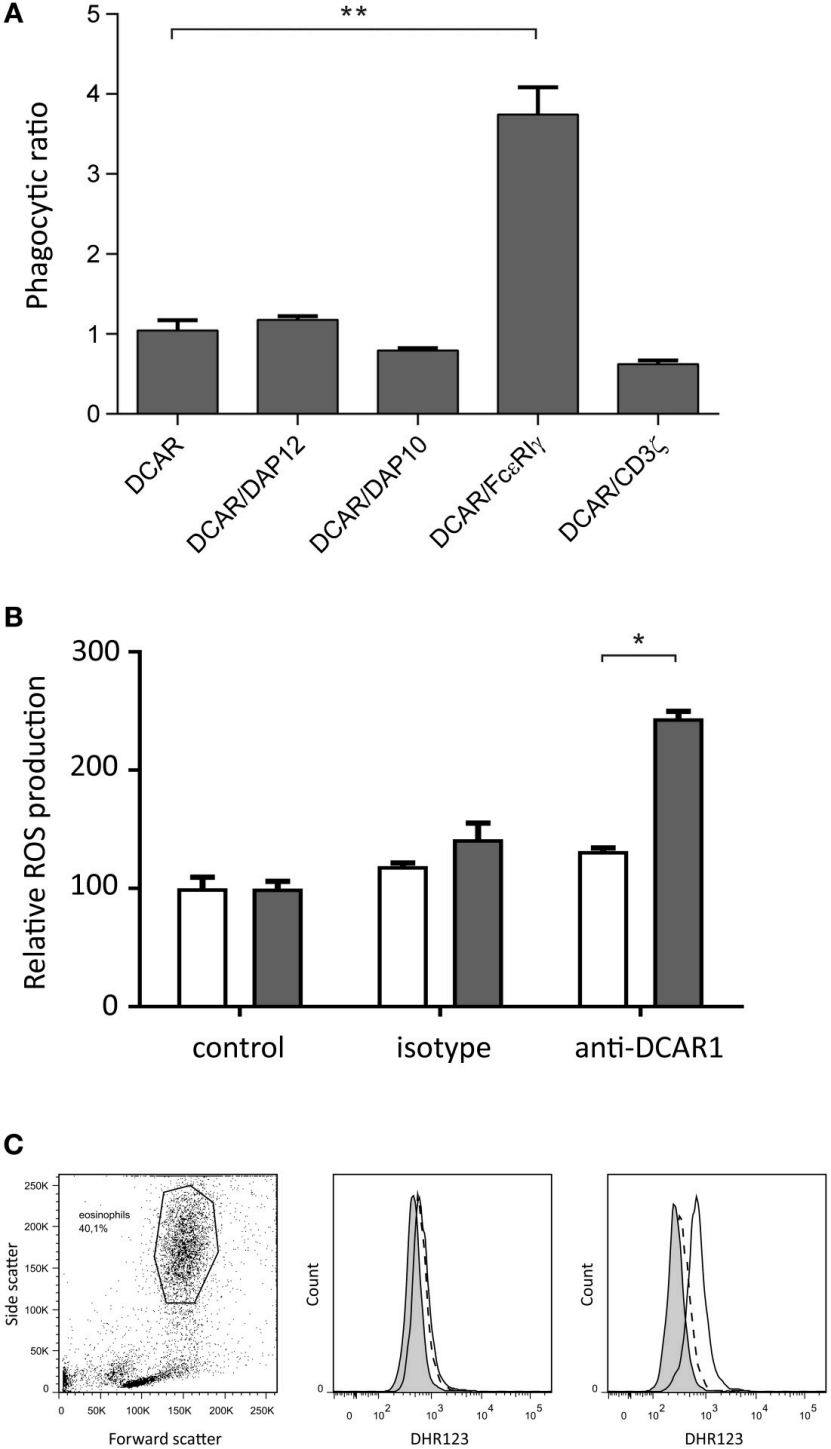
Rat DCAR1 activates effector functions. **(A)** Efficiency of phagocytosis of WEN41-coated microbeads by 293T cells transfected with DCAR1 alone, or together with the indicated adaptor proteins. **(B,C)** ROS production in eosinophils from DCAR1-expressing DA.APLEC^PVG^ rats (black columns) compared to DCAR1-deficient DA.NKC^PVG^ rats (white columns) following stimulation with mAb WEN41 or isotype control antibody. Mean values of triplicate samples are shown, with error bars indicating standard error of the mean. Data are representative of at least 3 independent experiments. Asterisks indicate significant differences ^*^p < 0.01, ^**^p < 0.0001.

We have previously shown that transfected 293T cells can mediate phagocytosis of antibody-coated beads (9). To investigate whether association of rat DCAR1 with FcεRIγ increased phagocytosis efficiency of antibody-coated beads, we transfected 293T cells either with DCAR1 alone or together with each of the four adaptors, and examined phagocytosis of beads coated with antibody against DCAR1. To correct for varying expression of DCAR1 on the cell surface, the ratio of cells with internalized beads to cells with only cell-surface beads was used as a measure of phagocytic efficiency. The efficiency of phagocytosis was not influenced by co-transfection with CD3ζ, DAP12, or DAP10, but phagocytosis was significantly higher in cells that had been co-transfected with FcεRIγ (Figure 7A), providing evidence that rat DCAR1 forms a functional activating signaling complex with FcεRIγ.

### Cross-Linking of DCAR1 on Eosinophils Mediates ROS Production

Since eosinophils showed the highest expression of DCAR1, we enriched the eosinophil population from peritoneal lavage. Following cross-linking of DCAR1 on the cell surface, eosinophils responded with a respiratory burst (Figures 7B,C), indicating that DCAR1 functions as an activating receptor also on primary cells.

## Discussion

As described above, association of the APLEC to autoimmunity has been demonstrated both in rodents and in man. In rats, the DA haplotype confers susceptibility to autoimmune disease. The causal APLEC gene has not been determined, but the DCAR1 gene is clearly interesting due to the early nonsense mutation in the *Dcar1* gene which prevents surface expression of the receptor in the DA strain (1). Studies on this receptor in the rat have been hampered by the lack of information about its expression. Here we describe the development of a monoclonal antibody that reacts with rat DCAR1, and we use this antibody to characterize the receptor in the rat.

In the first description of the DCAR receptors (mouse DCAR2), Kanazawa et al. reported strong expression in lung and spleen and weak in skin and lymph nodes (8). In the later report on mouse DCAR1, Kaden et al. restricted their investigations to blood, bone marrow and organized lymphoid tissues (7). As the expression profiles for mouse DCAR2 were based on RT-PCR, the present report is the first immunohistochemical study of DCAR1 expression profiles in diffuse mucosa associated lymphoid tissues. It is therefore noteworthy that by far the highest rat DCAR1 expression was found in the lamina propria of the gut and the peritoneal cavity, where expression was particularly seen on eosinophils. Whether the differences in tissue distribution between mouse DCAR2 (high in bronchus-associated lymphoid tissue—BALT, low in gut-associated lymphoid tissue—GALT) and rat DCAR1 (low in BALT, high in GALT), reflect species differences (mouse vs. rat), paralogue differences (DCAR2 vs. DCAR1), or technical differences (RT-PCR vs. immunohistochemistry) is unknown.

Rat DCAR1 was found to be expressed at low levels on myeloid cells in blood and unstimulated lymphoid tissues (spleen, thymus, and cervical lymph nodes—Figure 1, and data not shown), as also reported for mouse DCAR1 by Kaden et al. (7). Highest expression was seen on eosinophils, but we also saw DCAR1 expression on subsets of CD11b/c^hi^ MHC-II^hi^ cells that we presume to be DC. DCs are not as well defined in the rat as in the mouse or human, but two subsets of conventional DC have previously been identified in the spleen (14). These correspond to the P1 and P2 subsets we identified here (Figure 2A), and they most likely correspond to cDC1 and cDC2 subsets described in the mouse and human. DCAR1 expression was highest on the P2 (cDC2) subset. These cells have previously been shown to have a mixed Th1/Th2-polarizing ability, while the P1 (cDC1) population was strictly Th1-polarizing (14). CD103 is an inconsistent marker for DC in the mouse, with expression varying depending on the tissue, and on whether the DC are lymphoid-resident or migratory (17). It is unclear whether CD103 in the rat shows similar expression to the mouse, but it is conceivable that P2 and P3 populations (Figure 2A) are both cDC2, and that DCAR1 expression is limited to this DC subgroup. Alternatively, the P3 population could represent a distinct macrophage subset.

In the mesenteric lymph nodes, high expression of DCAR1 was seen on a population of cells with high side-scatter, suggestive of eosinophils. The presence of eosinophils in the lymph node is unusual, but eosinophils have been shown to be able to migrate to lymph nodes and present antigen to T cells in experimental models of allergic inflammation (18), and eosinophils can also be found in lymph nodes of asthma patients (19). The side-scatter of the cells we find in mesenteric lymph nodes is slightly lower than that of eosinophils from blood, and they express higher levels of MHC-II and CD4 than blood eosinophils. However, eosinophils are able to increase expression of both MHC-II and co-stimulatory molecules upon migration in to the lymph nodes (20). Unlike DC, eosinophils are unable to stimulate naïve T cells (21), but they may stimulate antigen-sensitized T-cells to amplify a Th2 response in the gut.

Polarization of peritoneal macrophages revealed that DCAR1 expression is highest on M2 macrophages (and virtually absent on M1 macrophages) but only on CD11b/c^lo^ macrophages. By analogy to mouse peritoneal macrophages (15), these probably correspond to a migratory population, and the expression of CCR7 on this population would support that claim. Why DCAR1 expression is seen on migratory macrophages and not resident macrophages is unclear, but mouse studies suggest that under inflammatory conditions this population becomes the dominant macrophage population in the peritoneum (15). Together with the expression of DCAR1 on cDC2 and eosinophils, our hypothesis would be that DCAR1 may play a role in type 2 immune responses, such as those involved in the immune response against protozoa or helminths. This is in contrast to the other activating receptors in the APLEC complex, MCL, Mincle and Dectin-2, which have been shown to be preferentially expressed on M1-polarized macrophages, and to be involved in the recognition of fungi, bacteria and mycobacteria (22–26). Indeed, mouse DCAR2 has recently also been shown to recognize phosphatidyl-inositol mannosides from mycobacteria, and to promote a Th1 response (27), while mouse DCAR1 triggering on CD8+ DC (cDC1) leads to the release of cytokines suggestive of Th1 induction (increased IL-12, decreased IL-10) (7). Thus rat DCAR1 diverges from the mouse DCAR receptors in regards to its expression pattern.

In the mouse, Kanazawa showed that DCAR2 (in his paper called DCAR) is an activating receptor associated with FcεRIγ (8). Close sequence similarities between the two mouse DCAR receptors has led to the assumption that DCAR1 is also activating, but there has been no formal proof of this. Here we show that in the rat DCAR1 can associate with the adaptor FcεRIγ, which mediates activating signals. Furthermore, we show that association with FcεRIγ leads to increased phagocytosis of antibody-coated beads, confirming that the DCAR1/ FcεRIγ complex generates an activating signal. We also demonstrate that activation of DCAR1 on eosinophils leads to the respiratory burst. In eosinophils, the production of ROS can play a role in destruction of parasites, but it may also play a role in the dampening of inflammation via the inactivation of endogenous damage-associated molecular patterns (DAMPs) (28). Other receptors in the APLEC region have been shown to have both endogenous (DAMP) and exogenous (PAMP) ligands, and the same is likely the case for DCAR1. Thus, DCAR1 may well play a role both in parasite defense and in inflammation resolution.

In mammals, *Dcar1* has so far only been identified in the rat and mouse genomes. In the duck, three genes encoding receptors with sequence similarity to the APLEC receptors have been reported. Two of these, named DCAR1 and−2, were predicted to be activating, and one, named DCIR, to be inhibitory (29). In phylograms, however, the duck receptor cluster with the Mincle branch, which by phylogenetic analyses seem to represent the oldest branch of the APLEC gene tree (1). Humans lack a *Dcar1*-ortholog, but instead contain another lectin-like receptor, CD303 (DLEC or BDCA-2) which is not found in rat and mouse (30). Based on sequence similarity, we have previously suggested that CD303 may represent the human structural ortholog of Dcar1 (1). CD303 is a marker for plasmacytoid dendritic cells in human. Using markers that define rat pDCs revealed a population that did not show any expression of DCAR1. In addition, the expression of DCAR1 on multiple myeloid populations is also distinct from the expression of CD303. It is therefore unlikely that DCAR1 and CD303 are functionally equivalent receptors, a conclusion also reached by Kaden et al. (7).

In summary, we show that DCAR1 is an activating receptor expressed on granulocytes and on subsets of cDCs and macrophages, but not on lymphocytes or pDCs. Expression is particularly pronounced on eosinophils, and is increased on M2-polarized macrophages, suggesting that DCAR1 may play a role in the immune response against protozoa or helminths.

## Ethics Statement

This study was carried out in accordance with the recommendations of The European Union's Directive 2010/63/EU on the protection of animals used for scientific purposes. The protocol was approved by the Norwegian Food Safety Authority.

## Author Contributions

MD and SF contributed to the conception and design of the study. MD, SF, and ED acquired funding. SF, MD, BN, and AL-P performed experiments and analyzed the data. MD wrote the first draft of the manuscript. RJ contributed to the resources. All authors contributed to manuscript revision, read and approved the submitted version.

### Conflict of Interest Statement

The authors declare that the research was conducted in the absence of any commercial or financial relationships that could be construed as a potential conflict of interest.

## Supplementary Material

Figure S1 Immunohistochemical staining of frozen sections of lung tissue taken from DA.APLEC^PVG^ or DA.NKC^PVG^ rats. Sections were stained with an antibody against MHC class II (OX6), which reveals macrophages and dendritic cells in the lung parenchyma. Staining with anti-DCAR1 antibody failed to reveal any positively-stained cells in the lung.

## References

[1] Flornes LM Bryceson YT Spurkland A Lorentzen JC Dissen E Fossum S. Identification of lectin-like receptors expressed by antigen presenting cells and neutrophils and their mapping to a novel gene complex. Immunogenetics. (2004) 56:506–17. 10.1007/s00251-004-0714-x 15368084

[2] Zelensky AN Gready JE. The C-type lectin-like domain superfamily. FEBS J. (2005) 272:6179–217. 10.1111/j.1742-4658.2005.05031.x 16336259

[3] Lorentzen JC Flornes L Eklow C Backdahl L Ribbhammar U Guo JP. Association of arthritis with a gene complex encoding C-type lectin-like receptors. Arthritis Rheum. (2007) 56:2620–32. 10.1002/art.22813 17665455

[4] Rintisch C Kelkka T Norin U Lorentzen JC Olofsson P Holmdahl R. Finemapping of the arthritis QTL Pia7 reveals co-localization with Oia2 and the APLEC locus. Genes Immun. (2010) 11:239–45. 10.1038/gene.2010.2 20200546

[5] Flytzani S Stridh P Guerreiro-Cacais AO Marta M Hedreul MT Jagodic M. Anti-MOG antibodies are under polygenic regulation with the most significant control coming from the C-type lectin-like gene locus. Genes Immun. (2013) 14:409–19. 10.1038/gene.2013.33 23784360

[6] Guo JP Verdrengh M Tarkowski A Lange S Jennische E Lorentzen JC. The rat antigen-presenting lectin-like receptor complex influences innate immunity and development of infectious diseases. Genes Immun. (2009) 10:227–36. 10.1038/gene.2009.4 19279651

[7] Kaden SA Kurig S Vasters K Hofmann K Zaenker KS Schmitz J. Enhanced dendritic cell-induced immune responses mediated by the novel C-type lectin receptor mDCAR1. J Immunol. (2009) 183:5069–78. 10.4049/jimmunol.0900908 19786536

[8] Kanazawa N Tashiro K Inaba K Miyachi Y. Dendritic cell immunoactivating receptor, a novel C-type lectin immunoreceptor, acts as an activating receptor through association with Fc receptor gamma chain. J Biol Chem. (2003) 278:32645–52. 10.1074/jbc.M304226200 12777403

[9] Lobato-Pascual A Saether PC Fossum S Dissen E Daws MR. Mincle, the receptor for mycobacterial cord factor, forms a functional receptor complex with MCL and FcepsilonRIgamma. Eur J Immunol. (2013) 43:3167–74. 10.1002/eji.201343752 23921530

[10] Westgaard IH Dissen E Torgersen KM Lazetic S Lanier LL Phillips JH. The lectin-like receptor KLRE1 inhibits natural killer cell cytotoxicity. J Exp Med. (2003) 197:1551–61. 10.1084/jem.20021253 12782717 PMC2193914

[11] Huh SH Do HJ Lim HY Kim DK Choi SJ Song H. Optimization of 25 kDa linear polyethylenimine for efficient gene delivery. Biologicals. (2007) 35:165–71. 10.1016/j.biologicals.2006.08.004 17084092

[12] Anjubault T Martin J Hubert FX Chauvin C Heymann D Josien R. Constitutive expression of TNF-related activation-induced cytokine (TRANCE)/receptor activating NF-kappaB ligand (RANK)-L by rat plasmacytoid dendritic cells. PLoS ONE. (2012) 7:e33713. 10.1371/journal.pone.0033713 22428075 PMC3302772

[13] Lobato-Pascual A Saether PC Dahle MK Gaustad P Dissen E Fossum S. Rat macrophage C-type lectin is an activating receptor expressed by phagocytic cells. PLoS ONE. (2013) 8:e57406. 10.1371/journal.pone.0057406 23468983 PMC3585393

[14] Voisine C Hubert FX Trinite B Heslan M Josien R. Two phenotypically distinct subsets of spleen dendritic cells in rats exhibit different cytokine production and T cell stimulatory activity. J Immunol. (2002) 169:2284–91. 10.4049/jimmunol.169.5.2284 12193693

[15] Ghosn EE Cassado AA Govoni GR Fukuhara T Yang Y Monack DM. Two physically, functionally, and developmentally distinct peritoneal macrophage subsets. Proc Natl Acad Sci USA. (2010) 107:2568–73. 10.1073/pnas.0915000107 20133793 PMC2823920

[16] Matsumoto M Tanaka T Kaisho T Sanjo H Copeland NG Gilbert DJ. A novel LPS-inducible C-type lectin is a transcriptional target of NF-IL6 in macrophages. J Immunol. (1999) 163:5039–48. 10528209

[17] Macri C Pang ES Patton T O'Keeffe M. Dendritic cell subsets. Semin Cell Dev Biol. (2017) 84:11–21. 10.1016/j.semcdb.2017.12.009 29246859

[18] Shi HZ Humbles A Gerard C Jin Z Weller PF. Lymph node trafficking and antigen presentation by endobronchial eosinophils. J Clin Invest. (2000) 105:945–53. 10.1172/JCI8945 10749574 PMC377484

[19] Cagnoni EF Ferreira DS Ferraz da Silva LF Nicoletti Carvalho Petry AL Gomes dos Santos AB Rodrigues Medeiros MC. Bronchopulmonary lymph nodes and large airway cell trafficking in patients with fatal asthma. J Allergy Clin Immunol. (2015) 135:1352–7. 10.1016/j.jaci.2014.08.021 25262462

[20] Duez C Dakhama A Tomkinson A Marquillies P Balhorn A Tonnel AB. Migration and accumulation of eosinophils toward regional lymph nodes after airway allergen challenge. J Allergy Clin Immunol. (2004) 114:820–5. 10.1016/j.jaci.2004.08.011 15480321

[21] van Rijt LS Vos N Hijdra D de Vries VC Hoogsteden HC Lambrecht BN. Airway eosinophils accumulate in the mediastinal lymph nodes but lack antigen-presenting potential for naive T cells. J Immunol. (2003) 171:3372–8. 10.4049/jimmunol.171.7.3372 14500630

[22] Sato K Yang XL Yudate T Chung JS Wu J Luby-Phelps K. Dectin-2 is a pattern recognition receptor for fungi that couples with the Fc receptor gamma chain to induce innate immune responses. J Biol Chem. (2006) 281:38854–66. 10.1074/jbc.M606542200 17050534

[23] Matsunaga I Moody DB. Mincle is a long sought receptor for mycobacterial cord factor. J Exp Med. (2009) 206:2865–8. 10.1084/jem.20092533 20008525 PMC2806465

[24] Yamasaki S Matsumoto M Takeuchi O Matsuzawa T Ishikawa E Sakuma M. C-type lectin Mincle is an activating receptor for pathogenic fungus, *Malassezia*. Proc Natl Acad Sci USA. (2009) 106:1897–902. 10.1073/pnas.0805177106 19171887 PMC2644135

[25] Miyake Y Toyonaga K Mori D Kakuta S Hoshino Y Oyamada A. C-type lectin MCL is an FcRgamma-coupled receptor that mediates the adjuvanticity of mycobacterial cord factor. Immunity. (2013) 38:1050–62. 10.1016/j.immuni.2013.03.010 23602766

[26] Rabes A Zimmermann S Reppe K Lang R Seeberger PH Suttorp N. The C-type lectin receptor mincle binds to *Streptococcus pneumoniae* but plays a limited role in the anti-pneumococcal innate immune response. PLOS ONE. (2015) 10:e0117022. 10.1371/journal.pone.0117022 25658823 PMC4319728

[27] Toyonaga K Torigoe S Motomura Y Kamichi T Hayashi JM Morita YS. C-type lectin receptor DCAR recognizes mycobacterial phosphatidyl-inositol mannosides to promote a Th1 response during infection. Immunity. (2016) 45:1245–57. 10.1016/j.immuni.2016.10.012 27887882

[28] Lotfi R Herzog GI DeMarco RA Beer-Stolz D Lee JJ Rubartelli A. Eosinophils oxidize damage-associated molecular pattern molecules derived from stressed cells. J Immunol. (2009) 183:5023–31. 10.4049/jimmunol.0900504 19794066

[29] Guo X Branton WG Moon DA Xia J Macdonald MR Magor KE. Dendritic cell inhibitory and activating immunoreceptors (DCIR and DCAR) in duck: genomic organization and expression. Mol Immunol. (2008) 45:3942–6. 10.1016/j.molimm.2008.06.014 18657864

[30] Dzionek A Sohma Y Nagafune J Cella M Colonna M Facchetti F. BDCA-2, a novel plasmacytoid dendritic cell-specific type II C-type lectin, mediates antigen capture and is a potent inhibitor of interferon alpha/beta induction. J Exp Med. (2001) 194:1823–34. 10.1084/jem.194.12.1823 11748283 PMC2193584

[31] Lobato Pascual A Molecular Characterisation of Activating Receptors in the Rat APLEC Gene Complex. Ph.D., University of Oslo (2014).

