# Supplementary figures and images for: Dendritic Cell Activating Receptor 1 (DCAR1) Associates With FcεRIγ and Is Expressed by Myeloid Cell Subsets in the Rat
Source: Front Immunol. 2019 May 10;10:1060. doi: 10.3389/fimmu.2019.01060 (PMC6522936; doi:10.3389/fimmu.2019.01060)

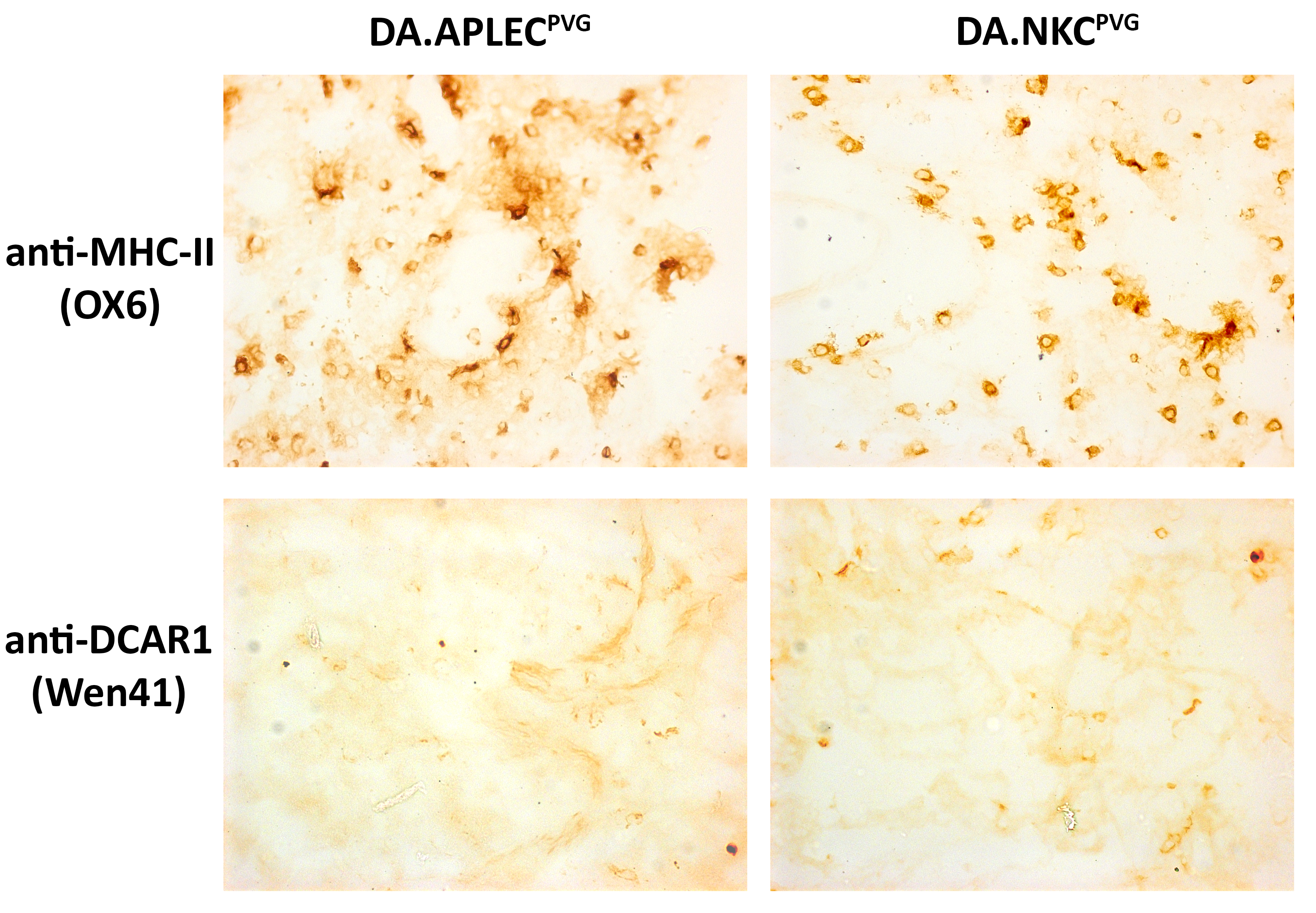

Supplement: Figure S1 — Immunohistochemical staining of frozen sections of lung tissue taken from DA.APLECPVG or DA.NKCPVG rats. Sections were stained with an antibody against MHC class II (OX6), which reveals macrophages and dendritic cells in the lung parenchyma. Staining with anti-DCAR1 antibody failed to reveal any positively-stained cells in the lung. [file Image_1.TIF]
